# Developmental study of rectum in broiler chicken: A stereological and morphometrical study

**Published:** 2016-03-15

**Authors:** Rahmat-Allah Fatahian Dehkordi, Poria Ghahremani

**Affiliations:** 1 *Department of Basic Sciences, Faculty of Veterinary Medicine, Shahrekord University, Shahrekord, Iran; *; 2 * DVM Student, Faculty of Veterinary Medicine, Shahrekord University, Shahrekord, Iran‎.*

**Keywords:** Broiler chicken, Developmental period, Morphometrical study, Rectum

## Abstract

The objective of this study was to investigate development of the weight and the morphological development of the rectum in broiler chickens. Twenty broiler chickens (Ross 308) were used in this experiment and they were 12, 20, 35 and 44 days of age. Samples from the rectum of chicks were sectioned in an unbiased manner and examined quantitatively using stereology. In addition, the weight of both body (BW) and rectum and also rectum weight as a proportion of BW, height and width of the villi and thickness of rectum wall were measured. The results revealed that the body and rectum weight were increased with age. The greatest rectum weight as a proportion of BW was observed on day 20. An increase in height and width of the villi during the study period were obtained. The increase was more significant on days 35 and 44 than on days 12 and 20 (*p* < 0.05). There were significant difference in thickness of tunica mucosa, submucosa and muscular layer of the rectum on day 20 compared to day 35 (*p* < 0.05). The increase in the volume density of the wall, tunica mucosa and tunica muscular was greater on day 20 than on day 35. However, significant differences were observed in volume density of this layers between days 35 and 44 compared to days 12 and 20 (*p* < 0.05).

## Introduction

It has been detected that the microscopic structure of the avian large intestine tract is affected by diet. Some studies have shown that the overall size of the gut and luminal surface area in some birds varies in relation to diet.^1^^-^^3^ A larger volume or surface area of the intestine presumably allows increasing further input in nutrients, which these foodstuffs can be digested and/or absorbed.^4^ Generally, the diluted diet causes amplification of the surface area due to villi. ^5^^-^^9^ Goldstein *et al*. showed that villus of large intestine is altered with diet in wild birds.^10^

In birds, the size and dimension of the digestive tract and consequently the digestion may be minimized.^11^^-^^13^ Therefore, intestinal luminal morphology is a basal function that is related to gut volume and may be reduced in Volant species.^4^

At the time of hatching, the development stage of the digestive tract, especially intestines determine the chicks' capability to consume feed.^2^ The colon was characterized by short luminal villi and goblet cells dispersed in the epithelium.^14^ In between and within species, large intestine includes a great morphometrical and functional variability. For instance, many familiar avian species, such as chickens and ducks, have individual large intestine, which aid in the digestion of nutrition and in water balance.^15^^,^^16^ The influx of water into large intestine can be reabsorbed through the epithelium of the large intestine tract to maintain hydration.^17^^-^^19^ This study was designed to understand the morphometrical and quantitative changes of rectum of the Ross broiler chickens with aging.

## Materials and Methods

**Tissue processing. **Twenty broiler chickens (Ross 308 breed) from the same batch were used in this study. Experiment was carried out in Shahrekord University, Iran. Chickens access to water and a standard ration was *ad libitum* (Starter: 32 MJ metabolizable energy (ME) per kg of diet, 230 g kg^-1^ crude protein (CP), Grower: 32 MJ metabolizable energy (ME) per kg of diet, 200 g kg^-1^ CP formulated to meet requirements for broilers.^20^

The birds were divided into four groups (n = 5): 12, 20, 35 and 44 day. The birds were immediately transported to laboratory for intestinal tissue processing. Body weight was obtained by a digital balance with accuracy 10^-3^. Birds were sacrificed by an overdose (65 mg mL^-1^) intra-peritoneal injection of sodium pentobarbital. The abdominal cavity was exposed and the lumen of the intestinal tracts was flushed using normal saline solution and intestines were removed. The rectum was cut and its fresh weight was calculated. The proximal, middle and distal segments of the rectum were dissected. Samples were fixed by immersion in buffered formalin fixative solution for 48 hr and then, samples were dehydrated and embedded in the paraffin. Paraffin-embedded blocks were cut at 5 µm thickness by microtome and in an unbiased style at 20 equally spaced intervals through the wax blocks. The tissue sections were dewaxed and were stained with Hematoxylin and Eosin (H&E) and were then studied under light microscope (Nikon, Düsseldorf, Germany). Photo-graphs were taken using a camera attached to microscope with maximum resolution. The villus height was measured from the top of the villus to the top of the lamina propria.

**Stereological analysis. **Cavalieri's Principle was used to estimate volume densities (*Vv*) of the rectum by point counting under light microscopy.^21^ For point counting, intersections between an eyepiece test grid and light micrographs were obtained. The number of intersections on the grid overlying the tissue component of the mucosa, submucosa, muscularis tunicae and also intestinal wall was counted for each sample. The ratio of points to the total number of the grid point was considered to be the volume density of each component, as in the following equation:

$\mathrm{Vv}=\frac{\mathrm{Pn}}{\mathrm{Pt}}$

where, *Vv* is the volume density of the tissue component, *Pn* is the number of intersections on the grid overlying the tissue component and *Pt* is the total number of points on the test grid.

The absolute volume of each component was calculated by the following formula:

$V=T\times \frac{a}{p}\times \sum_{i=l}^{m}\mathrm{pi}$

where, *T* is thickness of each slice, *a/p* is the area associated with each point of the test grid and *pi* is the number of points landing within each tracing.^22^

The surface areas (*SA*) of components were estimated using the following formula:^23^

$\mathrm{SA}=\frac{\mathrm{Vv}}{d}\times 4\times V$

where, *Vv* is the volume of the measured component by the Cavalieri Principle, *V* is absolute volume,* d* is the mean diameter of the component measured, (*d*: if a component was cut crossly, the short axis of its profile was measured). Data obtained were analyzed using the SPSS (Version 11.5; SPSS Inc., Chicago, USA). The statistical analysis was carried out by one-way ANOVA in level *p* < 0.05. All results are represented as mean ± SEM.

## Results

Body weights and rectum weights are presented in Table 1 showing the expected increase from 12 to 44 days post hatching (*p* < 0.05). There was significant increase in BW from 520.4 ± 38.0 g on day 12 to 2363.3 ± 201.0 g on day 44. The body weight gains were greater on days 35 to 44 than on days 12 to 20 (*p* < 0.05). Total rectum weights was increased more rapidly on 35 to 44 days than on 12 to 20 days, the slowest value of rectum growth was observed on 12 to 20 days. However, the weight of rectum was increased and reached its highest value on day 44. The rectum weight as a proportion of BW was greatest on day 20 for the age groups studied (*p* < 0.05).

**Table 1:** Body weights and colon morphometrics of chickens, fresh tissue basis

**Parameter**	**Age (Day)**			
	**12**	**20**	**35**	**44**
**Body weight (g)**	520.40 ± 38.00	742.70 ± 98.00	2014.60 ± 132.00	2363.30 ± 201.00
**Colon weight (g)**	2.04 ± 0.12	3.02 ± 0.24	4.15 ± 0.46	5.41 ± 0.35
**Colon weight/Body weight ratio**	38.60 (×10^-4^)	40.60 (×10^-4^)	20.60 (×10^-4^)	22.80 (×10^-4^)

**Fig. 1:**
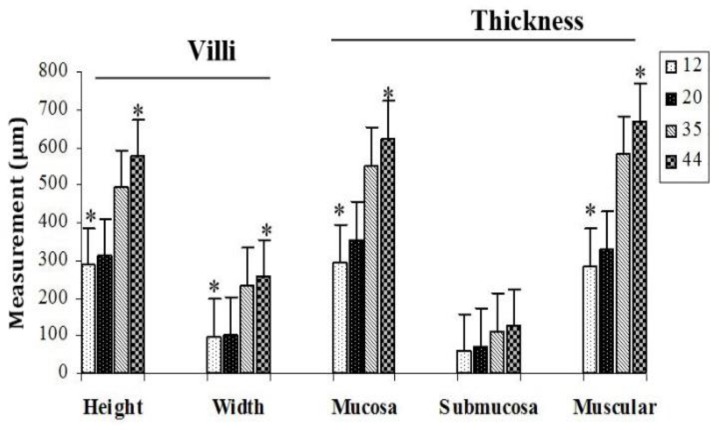
Micrometric analysis of villi height and width and thickness of the rectum different layers in chickens in age groups 12 to 44 days.

Results obtained from micrometric analysis of tunica mucosa, tunica submucosa and tunica muscularis of the rectum revealed a small increase in thickness between days 12 to 20 and also between days 35 to 44, however, a larger increase was observed between day 20 when compared to day 35 (*p* < 0.05), (Fig. 1). The villi height and width in rectum were increased with age, however, these changes were significant on days 35 and 44 than on days 12 and 20 (*p* < 0.05), (Fig. 1). 

* indicates significant difference among different days (*p *< 0.05).

**Fig. 2:**
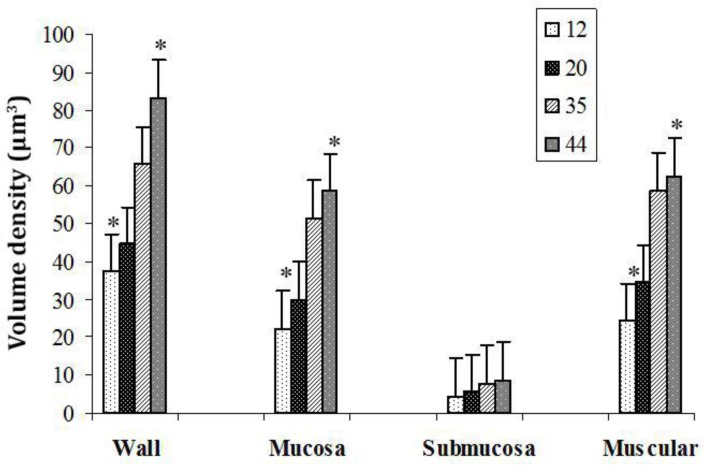
The volume density of the different layers of the rectum wall in chickens, in age groups 12 to 44 days.

* indicates significant difference among different days (*p *< 0.05).

**Fig. 3:**
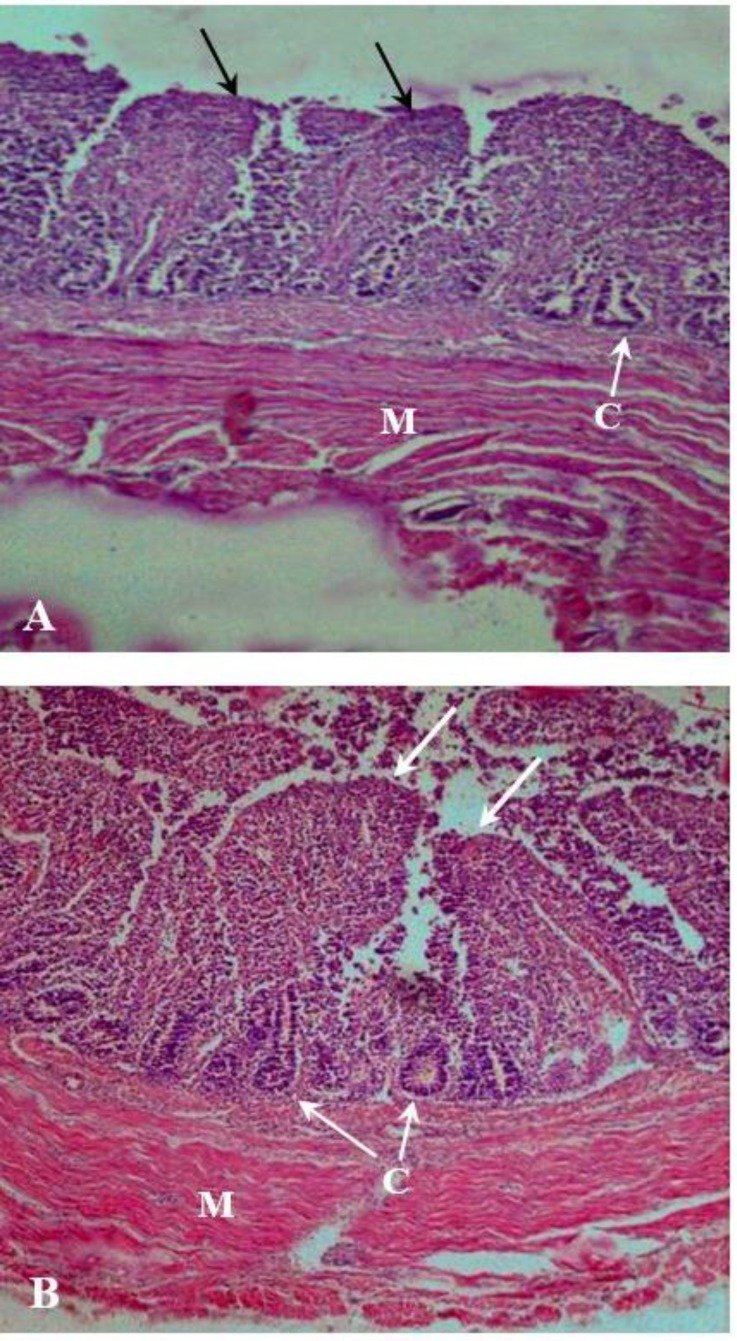
Photomicrographs of colon on 20 day (A) and 35 day (B) broiler chicken. These micrographs reveal the changes of villi on 20 day (black arrows) and 35 day (white arrows) broiler chickens rectum samples. Note the number of numerous villi and crypt (C) on 35 day and the 20 day in chicks; Muscular layer (M); (H & E, 200×).

The obtained results showed that volume density of various layers of rectum (Figs. 2 and 3), such as tunica mucosa, submucosa and muscular, gradually were increased in all age groups, however, this increase only was significant on days 35 and 44 than on days 12 and 20 (*p* < 0.05). 

## Discussion

Development of the intestinal tracts starts early in fetal life and quickly develop after birth and within this time a significant stride occur in the ontogeny of the intestinal tract in preparation of the neonate to encounter with nutrients.^24^ Our study revealed that as chicks grew, increase in weights of the both body and rectum occured. The size of digestive organs of birds was significantly affected by the amount and type of food taken by these birds.^25^^,^^26^ In a study by Jackson the intestinal dimensions showed positive relationship with body mass in 13 sea bird species investigated.^27^ In the present study, excluding one exception, the chicks' intestinal (rectum) weight was in reverse proportion (negative relation) to its BW. Reversed proportion was observed from day 20 to day 44 post hatching. In contrast, Barnes and Thomas on 18 species of ducks of the genus *Anatidae* reported a positive association between intestinal weights to the BW.^28^

In the present study, the thickness in tunica mucosa, tunica submucosa and tunica muscular of the rectum was increased with the age of the birds, from days 12 to 44. Also, the present results showed that thickness of the tnica mucosa and tunica submucosa on days 12 and 20 was slightly larger than the tunica muscularis in the same age group. There are a dense framework of fine collagen fibers within intstinal lamina propria which can affect this layer,^29^ therefore, it seems that the further increase of the tunica mucosa and tunica submucosa thickness than tunica muscularis at age groups of 12 and 20 days might be due to the more noticeable effect of this layer in intestine.

Design-based stereological methods permit efficient quantification of cells without bias from cell size, shape, orientation, or distribution. These quantitative advantages of the stereological procedures have been reported in some papers.^30^^,^^31^ In the present study, the villi height and width in rectum were increased with age and these results were similar to those of previous studies.^32^ Increase in villi height and width is matched by absorption function of the intestine, increase in digestion due to increase of absorptive surface area, expression of brush border enzymes and nutrient transport systems.^33^^,^^34^ Sklan, showed that the intestinal villi increase significantly in diameter and length during the first 7 to 10 days after hatching.^35^

The present study indicated that mean volume density of rectum wall was larger significantly in two age group 35 and 44 days compared to days 12 and 20. In goslings, it has been shown that volume density of intestinal villus wall was increased greatly from 4 to 21 post hatching.^34^ In two diet with low salt and high salt, Mayhew *et al*. reported a higher volume density in the part of the rectum wall (coprodeum wall) of hens that fed on a low salt diet compared to a high salt diet.^36^ However, There were differences in in morphometrical parameters of rectum samples. Values on day 44 were greatest than other age groups. The further transformation of rectum wall in broiler chicken was observed on day 20. 
